# Preterm Birth and Maternal Mood States: What Is the Impact on Bonding?

**DOI:** 10.3390/pediatric16010004

**Published:** 2024-01-05

**Authors:** Chiara Ionio, Giulia Ciuffo, Caterina Colombo, Olivia Melani, Maria Francesca Figlino, Marta Landoni, Francesca Castoldi, Francesco Cavigioli, Gianluca Lista

**Affiliations:** 1 Unità di ricerca sul Trauma, Dipartimento di Psicologia, Università Cattolica, 20123 Milano, Italyolivia.melani01@icatt.it (O.M.); mariafrancesca.figlino01@icatt.it (M.F.F.);; 2 Neonatologia Patologia e Terapia Intensiva Neonatale, Ospedale dei Bambini “Vittore Buzzi”, ASST Fatebenefratelli Sacco, Via Castelvetro 32, 20154 Milano, Italyfrancesca.castoldi@asst-fbf-sacco.it (F.C.); francesco.cavigioli@asst-fbf-sacco.it (F.C.); gianluca.lista@asst-fbf-sacco.it (G.L.)

**Keywords:** preterm birth, maternal mood states, mother–infant bonding

## Abstract

Preterm birth is a significant global health issue affecting millions of infants each year, with potential implications for their developmental outcomes. This study investigated the impact of preterm birth on maternal mood states during the early postpartum period and its subsequent effects on mother–infant bonding. Mothers of 90 preterm infants were involved in the assessment of maternal mood states, examined with the Profile of Mood States (POMS) questionnaire and the evaluation of mother–infant bonding, carried out through the Postpartum Bonding Questionnaire (PBQ). Contrary to expectations, there was no significant correlation between preterm birth characteristics and maternal mood states. On the other hand, significant correlations emerged between specific maternal mood states and the quality of mother–child bonding. More specifically, regression analyses showed that feelings of tension, anger, and confusion experienced by the mother tend to negatively affect the quality of her bond with her child. These findings emphasize the crucial role of maternal mental well-being in shaping the mother–infant relationship in the early postpartum period. The study highlights the importance of identifying and addressing maternal mood disorders to promote positive mother–infant bonding and child development, further underlining the need for comprehensive support and interventions for mothers of preterm infants.

## 1. Introduction

The World Health Organization (WHO) defines premature births as those that occur before the 37th week of pregnancy or less than 259 days after the first day of a woman’s last menstrual period [1]. Worldwide, an estimated 15 million babies are born prematurely each year, and preterm birth is the leading cause of infant death. In fact, 35% of newborns who are less than one month old die as a result of premature birth and its consequences [2]. In recent years, mortality rates have steadily decreased thanks to technological and scientific advances in neonatal and perinatal care that enable the survival of very low birth weight (VLBW) or even extremely low birth weight (ELBW) infants [3]. While this is positive news, prematurity is a significant risk factor for developmental disorders, which are more likely to occur with severe prematurity [4]. Indeed, the immature brain of preterm infants at birth is susceptible to particular forms of brain injury that are strongly correlated with adverse neurodevelopmental outcomes [5]. A lower IQ, lower executive skills, and a lower processing speed are some of the consequences associated with very premature birth [6], as is a higher likelihood of developing behavioral problems such as attention deficit hyperactivity disorder (ADHD) [7]. In addition, premature births often pose a risk not only to the survival and future development of the child, but also to the parents and, above all, to the physical and mental well-being of the mother. This is because the nine-month gestation period is not only important for the development of the child, but also for the woman’s psychological adjustment to motherhood and the development of ideas about the child [8]. An abrupt interruption of this process means that premature births are often perceived as stressful, unexpected, and therefore potentially traumatic events [9]. After giving birth, mothers who have given birth prematurely often feel separated from their child, have feelings of maternal inadequacy, a loss of parental role, and temporal abandonment [10]. Premature births can also trigger feelings of guilt for not being able to carry the baby to full term and for not being able to meet their babies’ needs after birth because they had to recover in the neonatal intensive care unit (NICU) [11]. It has been shown that women who experience preterm labor are at a higher risk of developing symptoms of anxiety, depression, and even post-traumatic stress disorder (PTSD) [12]. More specifically, anxiety symptoms can occur during pregnancy but usually peak during labor [13]. They can occur in all women but are much more pronounced following preterm labor [14]. In most cases, anxiety symptoms persist after infants are discharged from the NICU [15], although some variables, such as the presence of a supportive partner and the perception of good social support, tend to limit this risk [16]. Depressive symptoms are also common after birth [17], especially in preterm infants, 40% of whom suffer from this mood disorder [18]. Moreover, the risk of developing these symptoms seems to increase inversely proportional to the degree of prematurity of the child, so that the incidence rate is even higher in mothers with very premature infants weighing less than 1500 grammes [18]. More specifically, the incidence rate is up to 18 times higher in mothers of VLBW infants than in women who had a full-term birth [19]. This is attributed to the perception of the child’s fragility and fear for their life, where parents have limited room for intervention [20]. In addition, the neonatal intensive care unit (NICU) environment can be a source of stress and cause mothers to experience emotional crises [21]. Mothers of premature babies need to build a relationship with their baby in the NICU. The bright light, the presence of machines and monitors, as well as the unsafe living conditions of the children staying there, seem to be associated with the occurrence of depressive symptoms in the mothers [22]. In this context, the role of the father seems to act as a protective factor against the occurrence of depressive symptoms in the mother [23]. Another condition that jeopardizes the mental health of mothers is the possible development of the typical symptoms of post-traumatic stress disorder, which can occur after the perception of a particularly traumatic birth [24]. PTSD is a disorder that can affect all women, but those who give birth prematurely appear to be at higher risk of developing it [25]. What has been reported so far is worrying for clinicians, because maternal mental health is closely linked to the early relationship with the child, also known as attachment, and the resulting psychological and physical development of the child [26]. Parent–child attachment refers to an emotional, behavioral, cognitive, and neurobiological connection between a parent and their child [27]. It is a parent-driven process which can continue to evolve throughout the child and parent’s life, but to be adaptive, it should remain enduring, committed, and engaged [28]. Mother–infant bonding starts when the child is conceived and gradually strengthens throughout pregnancy and following birth through physical touch and skin-to-skin practices [29]. However, the latter appears to be challenging in the case of preterm children, whose hospitalization is an obstacle to the implementation of physical touch and closeness. In addition to this, many studies have shown that mood states such as anxiety, depression, or PTSD, often result in mothers being less sensitive to their child’s needs, which could further compromise the quality of their bonding [30,31]. When this occurs, the child’s short-term and long-term emotional development are at risk [32]. In the first case, babies whose mothers have symptoms of anxiety, depression, or PTSD show more signs of stress and experience a higher level of arousal. At one and two years of age, they tend to show less cognitive and emotional skills, which often continue to lack through infancy and adolescence, exposing them to a higher risk of developing behavioral issues [33,34]. In light of these premises, previous studies have investigated the relationship between maternal mood and bonding in mothers and preterm infants [35]. However, the results are contradictory. For example, Khemakhem et al. demonstrate that preterm birth appears to influence the psychological state of mothers and their interactions with their child [26]. In contrast, in the study by Bieleninik et al., the results did not show significant relationships between postpartum maternal bonding and maternal mental health (depression and anxiety) [36].

The present study sets two objectives: (i) to understand whether preterm birth has a negative impact on the mother’s mental health, and (ii) to analyze if maternal psychological illness compromises the quality of mother–infant bonding. From evidence which has emerged in the current literature [37], our first hypothesis is that preterm birth does expose women to a greater chance of developing mood disorders during the postpartum phase [38]. In addition, we hypothesized that experiencing a mood disorder postpartum might negatively impact the quality of mother–child bonding [39].

## 2. Materials and Methods

### 2.1. Data Collection

The present study is part of a broader longitudinal research project that started in 2013 in collaboration with CRIdee (Department of Psychology, Catholic University of the Sacred Heart, Milan, Italy) and the NICU and Obstetric department of the Buzzi Children’s Hospital in Milan (Italy). The research follows preterm infants from birth to 7 years of age. It was approved by the Ethics Committee of Azienda Ospedaliera, Istituti Clinici di Perfezionamento, Milano (protocol number: 1171, 11 December 2012). The aim of this broad study was to investigate, from a longitudinal perspective, the impact of preterm birth on child development while dedicating particular attention to the parental couple and to the effects and consequences that this event has on the triad. For this reason, families were followed in two phases: the first one began at birth, was intermediated by an assessment at three months of age, and ended with the child’s first birthday. The second phase of analysis began at age 4 and continued through annual observations until the premature child turned 7. The present paper focuses on the first phase of the longitudinal research and takes place within the first two weeks following childbirth. During this time, parents who had premature babies were approached and were introduced to the project. Once they agreed to participate, the parents both signed an informed consent form and privacy statement. On the same occasion, they were also given the Profile of Mood States Questionnaire and the Postpartum Bonding Questionnaire to fill out.

### 2.2. Participants

The mothers of 90 children who were born preterm between 2013 and 2019 participated in the present study. Inclusion criteria were having a gestational age <30 weeks and 6 days and birth weight <1250 g. Exclusion criteria were congenital anomalies, severe sensory impairment, severe brain injury or other neurological complications, and parents’ lack of Italian language skills. See Table 1 for demographic characteristics (Table 1).

### 2.3. Measures

Profile of Mood States (POMS) [40]

The Profile of Mood States (POMS) is a useful tool for identifying and quantifying particular affective states. The POMS is a self-report questionnaire that measures six factors and as many mood states, from anxious tension to depression and a sense of disorientation. Furthermore, the test provides a sensitive measure of the effects of various stressful conditions, whether they concern the subject’s life or are induced experimentally. The test consists of 58 adjectives that define six factors: Tension–Anxiety (factor T), Depression–Dejection (factor D), Aggression–Anger (factor A), Vigor–Activity (factor V), Tiredness–Indolence (factor S), and Confusion–Bewilderment (factor C). These adjectives are presented to the subject, who will have to choose the intensity with which she has been affected by that mood state in the last week. This allows the individual’s typical reactions to be evaluated over a fairly long period of time, but it also allows any change in the woman’s mood state to be highlighted. The intensity of the feeling is quantified using a 5-step Likert from 0 = not at all to 4 = very much [41]. Previous studies have shown that Cronbach’s alpha ranges from 0.84 to 0.94 for the POMS subscales and is 0.94 for the total score [42].

Postpartum Bonding Questionnaire (PBQ) [43]

The Postpartum Bonding Questionnaire (PBQ) is a tool whose aim is to measure the quality of parental–child bonding and identifying if there are any disturbances in the relationship. This questionnaire is useful for identifying difficulties that affect the mother–child bond. It is made up of 25 items divided into 4 scales: General relationship (12 items), Rejection and pathological anger (7 items), Anxiety for the child and care (4 items), and Imminent abuse or risk of abuse (2 items). The four scales are made up of items that describe some of the ways in which the parent might approach and relate to their child. The mother or father who responds to the questionnaire is required to honestly indicate how often they experience what is reported in each item using a 6-step Likert scale that ranges from 0 = never to 5 = always, with a maximum achievable score of 125. Higher scores indicate a higher degree of bonding difficulties. The authors suggested using a threshold value equal to 26 to signal the presence of the disorder and equal to 40 to indicate the potential for there to be a serious disorder. The individual scales also have specific threshold values, useful for identifying potential difficulties. In the case of the General Relationship scale, the threshold value is 12; for the Rejection and Pathological Anger scale, it is 17; for the Anxiety for the child and care scale, it is equal to 12; and for the Imminent Abuse or Risk of Abuse scale, it is 3. Subsequent studies have reported internal consistencies (Cronbach’s α) varying from 0.76 to 0.87 for the scale, 0.76 to 0.79 for the impaired bonding subscale, 0.63 to 0.75 for the rejection and anger subscale, 0.34 to 0.64 for the anxiety about care subscale, and 0.20 to 0.36 for the risk of abuse subscale [44,45,46,47].

### 2.4. Statistical Analysis

SPSS Statistics version 27.0 was used to analyze the data. Firstly, descriptive analyses were performed to examine the demographic characteristics of the sample. To investigate maternal mood states, we converted the raw scores of the POMS into T-values, with a mean of 50 and a standard deviation of 10, based on the Rasch person parameters. Descriptive statistics of the POMS and PBQ were performed. Correlation analyses were conducted to study the bivariate associations among all included variables. Once the association between the POMS and PBQ was verified, regression analyses were performed.

## 3. Results

### 3.1. Mothers’ Mood Profile after Preterm Birth

To verify our first hypothesis, which questioned whether preterm birth exposed mothers to a greater chance of developing negative mood states postpartum, we conducted descriptive statistics of the POMS questionnaire. The said analysis shows that the values of Tension–Anxiety, Depression–Dejection, Aggression–Anger, Vigor–Activity, and Confusion–Bewilderment are all within the average range, with scores that stay below the average value of T = 50, as listed in Table 2. The only index which appears to be above average is Tiredness–Indolence, where the average score obtained by the mothers of our sample is 55.17 ± 11.725. The results of the scales of the PBQ show that the values are all within the average range, with scores that did not exceed the reference threshold values.

Furthermore, the correlation analysis did not show a significant correlation between the POMS scales and characteristics of preterm birth of gestational age, birth weight, Apgar index, and days of hospitalization. This implies that, based on the results of the present study, there does not seem to be a bidirectional association between preterm birth and maternal mood states. The only index which resulted in being slightly correlated to the POMS scale of Depression–Dejection is the Apgar value at five minutes from birth (Apgar 5′; r = 0.267; *p* < 0.05). However, this correlation appears to be very weak and therefore is not particularly explicative. See Table 3 for correlation.

### 3.2. Maternal Mood States and Bonding

Moving into our second goal, the correlation analysis shows multiple significant correlations between maternal mood states as measured by the POMS questionnaire and the quality of maternal–child bonding as measured by the PBQ test. More specifically, the scale of General relationship measured by the PBQ is positively correlated to the POMS scales of Confusion–Bewilderment (r = 0.431; *p* < 0.01), Tension–Anxiety (r = 0.222; *p* < 0.05), Depression–Dejection (r = 0.289; *p* < 0.01), and Aggression–Anger (r = 0.367; *p* < 0.01). Significant correlations also emerged between the PBQ scale of Rejection–Pathological anger and the POMS scales of Tension–Anxiety (r = 0.291; *p* < 0.01), Confusion–Bewilderment (r = 0.343; *p* < 0.01), and Vigor–Anger (r = −0.242; *p* < 0.05). Lastly, a significant correlation was found between the PBBPQ scale of infant-focused anxiety and the index of tension (r = 0.238; *p* < 0.05) as measured by the POMS questionnaire. No significant correlation was found between the scale of Imminent abuse or risk of abuse and any of the scales measured by the POMS (Table 4).

To further understand the causality between the correlations that emerged, we also conducted regression analyses. Firstly, regression analyses show that the PBQ scale of General relationship is in fact predicted by all variables with which it had shown a positive correlation, except for the POMS scale of Depression–Dejection. More specifically, the POMS scale of Tension/Anxiety (β = −0.469; *p* = 0.010; R^2^ = 0.222); POMS scale of Aggression/Anger (β = 0.317; *p* = 0.042; R^2^ = 0.222); and POMS scale of Confusion/Bewilderment (β = 0.512; *p* = 0.002; R^2^ = 0.222) are variables which compromise the quality of bonding, causing its impairment. On the contrary, no significant regression models were found between the POMS scale of Tension/Anxiety and the scale of Aggression/Anger (β = 0.92; *p* = 0.549; R^2^ = 0.189). Instead, the PBQ scale of Rejection/Pathological anger is significantly predicted by Confusion/Bewilderment (β = 0.324; *p* = 0.035; R^2^ = 0.189) and Vigor/Activity (β = −0.325; *p* = 0.013; R^2^ = 0.189). Lastly, the regression analysis shows that Confusion/Bewilderment does, in fact, predict the infant-focused PBQ scale of Anxiety for child and care (β = 0.238; *p* = 0.025; R^2^ = 0.046). The results are presented in Table 5.

## 4. Discussion

The results of the correlation and regression analyses allow us to comment on the objectives and hypothesis that we formulated above. Regarding our first objective, it was hypothesized, based on the present literature, that there could be a correlation between preterm birth and maternal mood disorders [37]. However, contrary to our expectations, this hypothesis was not supported by our research data, which did not find significant correlations between the characteristics of preterm birth and mood states as measured by the POMS questionnaire. Moreover, the scores obtained by mothers on the POMS test were in line with the average scores obtained by the normative sample. Although this result is in contrast with our hypothesis, it does find support in the literature, which reports conflicting data on whether or not there might be a correlation between preterm birth and alteration of maternal mood in the postpartum period. On the one hand, some authors [48,49] argue that the likelihood of developing a maternal mood disorder following preterm birth is high due to the potentially traumatic nature of this type of birth. That said, although this possibility exists, some authors have also documented the opposite, reporting a similar perception of birth positivity among mothers of preterm and full-term babies [50]. This could be due to multiple concomitant variables at the time of preterm delivery, which include a lower number of follow-up visits that mothers receive during labor as well as being granted greater privacy given that premature births could potentially become emergency situations [51]. During the postpartum period, the presence of adequate maternal mood states could also be due to several aspects. Firstly, within the population of women who give birth preterm, there is a high percentage of mothers who have become such after having struggled to conceive or through medically assisted procreation, which, in fact, we know increases the chances of having a preterm birth [52]. Therefore, for many women, the moment of birth and acquisition of motherhood is so intensely awaited and desired that it might outweigh potential negative feelings resulting from prematurity [53]. Furthermore, it is worth mentioning that the moment of data collection is also important in understanding maternal mood states, which tend to be inconsistent in the postpartum period. In the present research, the questionnaires were completed by mothers within the first two weeks of delivery. This is a significant finding, as this period coincides with the child’s hospitalization in the NICU, a protected place that, despite its complexity, could reassure mothers about the adequate care of their children and thus reduce the prevalence of negative feelings [54]. For this reason, it is possible that an assessment carried out a few months after childbirth would have given a different picture, since it would also take the period following hospitalization into account, as well as maternal mood during the latter. In addition to this, the impact that the quality of hospital service and neonatal intensive care management have on maternal mood cannot be overlooked. It has been widely documented that some practices that can be implemented in the NICU, such as skin-to-skin and kangaroo care, are conducive to maternal well-being. The first technique was found to be related to a significant reduction in depressive symptoms experienced by mothers in the weeks following childbirth [55]. Breastfeeding also appears to be a protective factor for mothers, who report a sense of satisfaction and well-being in being able to provide for the physiological needs of their hospitalized child [56]. Furthermore, the presence of a figure who provides psychological support to mothers of preterm babies is an important resource that can help them process what they are experiencing and recognize, to counteract, the onset of negative mood states [57]. Considering this, it is easy to understand how the implementation of these techniques and a focus on maternal well-being within neonatal intensive care units can also be a protective factor against the onset of negative mood states.

The second objective of our research was aimed at understanding whether the presence of negative maternal mood states was correlated, and potentially predictive, of an impairment of the quality of bonding between mother and child. Correlation analyses illustrate that there is indeed an association between some maternal mood states and impaired bonding. What emerged is consistent with research that identifies maternal mood as a fundamental variable in the definition of the bonding relationship with the child [58]. Starting from the present study’s data, it should be remembered that tension, anger, and confusion experienced by the mother were predictive of the quality of bonding. This implies that feelings of anxiety, aggression, and bewilderment impact the mother–child relationship in the postpartum period. This is in line with the fact that bonding is a process driven by the parent, who has the responsibility of structuring it adaptively [28]. Daglae and Nur demonstrated that high anxiety levels negatively affect mother–baby bonding in the postpartum period [56]. Furthermore, Britton also found that anxious mothers were less sensitive, less responsive, and showed less competence in parenting [59]. If a mother shows reduced sensitivity, due to feelings of anxiety, hostility, or confusion, she might not be as able to build a strong bond with the baby, which is important because it responds to the child’s need for protection and closeness. Previous studies have shown that mothers who feel intense anger or hostility are less responsive towards their newborn, with whom they tend to limit physical and verbal manifestations of affection and closeness [60]. Such feelings of anger, according to the findings of the current study, could also be caused by a lower sense of vigor and a greater sense of confusion. In fact, as we can recall, the regression analysis highlighted a predictability between the variables investigated by the POMS questionnaire and anger assessed by the PBQ. Therefore, women who perceive a strong sense of tiredness and fatigue which, on the other hand, are inherent in the maternal role in the postpartum period, could find themselves experiencing greater feelings of anger and hostility, as could mothers who perceive a state of confusion that is greater than what they expected.

## 5. Strengths and Limitations

This study has several limitations. Firstly, only a few aspects of prematurity were considered in the search for a possible correlation between preterm birth and maternal mood disorders. Indeed, some important socio-relational variables, such as the presence of other children to take care of and the presence of single or multiple births, were not investigated in this research and would most likely impact the studied constructs. Also, the lack of consideration of the paternal role is a limitation of this study, which only focuses on the maternal figure. Future studies should investigate the implications of preterm birth on paternal mood states, as well as consider their impact on father–infant bonding. Furthermore, the presence of a supportive partner is one of the possible moderators/mediators of the impact of preterm birth on mother–child bonding and therefore should be analyzed in regard to the latter dyad as well. Another aspect worth mentioning is the short-term morbidity of preterm neonates, which may impact maternal mood states and therefore should be considered in the future. Moreover, the present study is part of a longitudinal project that evaluated the parental perceptions and development of preterm infants over several years through follow-up visits. In this case, the choice was made to only evaluate mother’s perceptions in the immediate postpartum. Future studies should consider a longer time frame to investigate the long-term impact of prematurity. It may also be useful to administer the same questionnaires following the child’s discharge. This is a moment which is often overlooked but that may present different challenges which parents have to face on their own for the first time since birth. Lastly, longitudinal studies are needed to generalize the results.

## 6. Conclusions

Contrary to what was hypothesized, no significant mood disturbances emerged within our sample of mothers two weeks postpartum. This could be due to several aspects, including the ability of the medical, obstetric, nursing, and psychological staff of the V. Buzzi hospital to adequately support mothers in the critical moments following childbirth. The second research objective, on the other hand, confirmed our hypotheses by revealing a significant correlation between the presence of certain maternal mood states and an impairment of bonding. More specifically, tension, anger, and confusion experienced by mothers seems to compromise their bonding with the child. Mothers who experience greater confusion or a lack of vigor also tend to foster greater feelings of anger; and finally, a greater state of confusion will predict a stronger sense of child-focused anxiety. These results underline that preterm birth is not necessarily a cause for negative maternal mood states, which can be prevented by a series of factors, allowing women a happy transition to motherhood. However, in circumstances where mothers do experience negative mood states, these must be carefully evaluated and addressed in a timely manner in order to prevent the possible short- and long-term impairment of the bonding with her child.

## Figures and Tables

**Table 1:** Characteristics of the sample.

Characteristic		n	%
Gender	female	50	55.6
	male	40	44.4
Type of pregnancy	single	45	50
	multiple	45	50
Type of twin pregnancy	monochorionic twins	30	55.6
	dichorionic twins	15	16.7
	Mean	SD	Range
Age of mother	36.58	5.065	23–44
Gestational age	28.61	2.402	23–34
Birth weight (g)	1092.56	306.889	430–1957
Apgar 1′	5.72	1.485	1–9
Apgar 5′	7.77	1.257	2–10
Days of hospitalization	65.02	24.619	19–131

**Table 2:** Means and standard deviations.

Variables	Mean	SD	Range
POMS Indices			
Tension–Anxiety	48.01	10.090	34–69
Depression–Dejection	48.74	9.509	3–71
Aggression–Anger	47.57	10.201	4–75
Vigor–Activity	46.76	12.136	26–75
Tiredness–Indolence	55.17	11.725	4–72
Confusion–Bewilderment	48.90	12.002	3–72
PBQ Indices			
General relationship	3.17	3.062	0–15
Rejection/Pathological anger	1.48	1.909	0–9
Anxiety for child and care	3.08	2.487	0–9
Imminent abuse or risk of abuse	0.74	1.204	0–5

Note: POMS: Profile of Mood States. PBQ: Postpartum Bonding Questionnaire.

**Table 3:** Correlation analysis between POMS scales and characteristics of preterm birth of gestational age, birth weight, Apgar index, and hospital days.

	POMS—Tension/Anxiety	POMS—Depression/Dejection	POMS—Aggression/Anger	POMS—Vigor/Activity	POMS—Tiredness/Indolence	POMS—Confusion/Bewilderment
Gestational age	−0.188	0.039	−0.054	0.063	−0.083	−0.132
Birth weight	−0.146	0.072	0.048	−0.072	−0.023	−0.094
Apgar 1′	0.020	0.179	0.123	0.122	0.029	0.071
Apgar 5′	0.064	0.267 *	0.163	0.029	0.081	0.114
Hospital days	0.126	0.057	−0.004	−0.076	0,100	0.159

* *p* < 0.05.

**Table 4:** Correlation analysis between POMS scales and PBQ scales.

	POMS—Tension/Anxiety	POMS—Depression/Dejection	POMS—Aggression/Anger	POMS—Vigor/Activity	POMS—Tiredness/Indolence	POMS—Confusion/Bewilderment
PBQ—General relationship	0.222 *	0.289 **	0.367 **	−0.075	0.181	0.431 **
PBQ—Rejection/Pathological anger	0.291 **	0.178	0.182	−0.242 *	0.073	0.343 **
PBQ—Anxiety for child and care	0.099	0.036	0.144	−0.049	0.044	0.238 *
PBQ—Imminent abuse or risk of abuse	0.149	0.106	0.077	−0.161	0.093	0.130

* *p* < 0.05, ** *p <* 0.001.

**Table 5:** Significant linear regression between PBQ and POMS subscales.

Dependent Variable	Predictor	β	*p*	R^2^
PBQ—General relationship	POMS—Tension/Anxiety	−0.469	0.010	0.222
	POMS—Aggression/Anger	0.317	0.042	0.222
	POMS—Confusion/Bewilderment	0.512	0.002	0.222
PBQ—Rejection/Pathological anger	POMS—Confusion/Bewilderment	0.324	0.035	0.189
	POMS—Vigor/Activity	−0.325	0.013	0.189
PBQ—Anxiety for child and care	POMS—Confusion/Bewilderment	0.238	0.025	0.046

## Data Availability

The dataset is not publicly available due to the hospital’s privacy policy.
